# The association between childhood hearing loss and self-reported peer victimisation, depressive symptoms, and self-harm: longitudinal analyses of a prospective, nationally representative cohort study

**DOI:** 10.1186/s12889-022-13457-6

**Published:** 2022-05-25

**Authors:** Emma Butcher, Mario Cortina-Borja, Carol Dezateux, Rachel Knowles

**Affiliations:** 1 Population, Policy and Practice Research and Teaching Department, UCL Great Ormond Street Institute of Child Health, London, UK; 2 Wolfson Institute of Population Health, Barts and the London School of Medicine and Dentistry, Queen Mary University of London, London, UK

**Keywords:** Child, Depressive symptoms, Cohort studies, Mental health, Hearing loss, Self-harm, Peer victimisation

## Abstract

**Background:**

Childhood hearing loss (HL) predicts poor mental health and is associated with a higher risk of communication difficulties. The relationship of childhood HL with specific types of poor mental health (such as depressive symptoms or self-harm) and peer victimisation remains unclear.

**Methods:**

We analysed data from the Millennium Cohort Study (MCS), a prospective observational cohort study of children living in the UK at age 9 months and born between 2000 to 2002. Data were available on the children and their families at ages 9 months, then at 3, 5, 7, 11, and 14 years.

Participants were 10,858 singleton children with self-reported data on peer victimisation, depressive symptoms, and self-harm at age 14 years. Multivariable logistic regression models were fitted to estimate odds ratios (OR) for HL with peer victimisation, depressive symptoms, and self-harm. HL presence was examined in terms of any HL between ages 9 months and 14 years, as well as by HL trajectory type (defined by onset and persistence). Analyses were adjusted for potential sources of confounding, survey design, and attrition at age 14 years. Interactions between sex and HL were examined in each model and multiple imputation procedures used to address missing data.

**Results:**

Children with any HL had increased odds of depressive symptoms (OR: 1.32, 95% CI: 1.09–1.60), self-harm (1.41, 1.12–1.78) and, in girls only, peer victimisation (girls: 1.81, 1.29–2.55; boys: 1.05, 0.73–1.51), compared to those without HL. HL with later age at onset and persistence to age 14 years was the only trajectory associated with all outcomes.

**Conclusions:**

Childhood HL may predict peer victimisation (in girls), depressive symptoms, and self-harm. Further research is needed to identify HL trajectories and methods to facilitate good mental health in children with HL.

**Supplementary Information:**

The online version contains supplementary material available at 10.1186/s12889-022-13457-6.

## Introduction

Hearing loss (HL) in childhood is associated with poor mental health [1, 2]. This may be due to a higher risk of language and communication difficulties [3], peer victimisation or bullying, where bullying is a specific form of victimisation in which there is an imbalance of power between the bully and the victim [4]. If so, interventions addressing victimisation and communication skills could improve mental health in children with HL.

Studies investigating specific types of poor mental health associated with HL have produced conflicting findings over whether risk of depressive symptoms is higher in children with HL [5–17]. Thus, it is not clear whether risk of depressive symptoms is unaffected by HL or whether there are particular groups of children with HL at increased risk of depressive symptoms. This hinders ability to identify children at risk of depressive symptoms and develop effective interventions.

There is also a lack of knowledge about the risk of peer victimisation [4] and self-harm associated with HL. A review of HL and peer victimisation concluded that available studies suggest, but do not confirm, that HL is associated with peer victimisation. Stronger conclusions could not be drawn due to the limited number of studies and methodological shortcoming of available studies [4]. Furthermore, previous studies on mental health and HL often focussed on permanent HL [3] and did not consider the influence of age at onset and persistence of HL. These factors may influence risk of peer victimisation and depressive symptoms, through their impact on communication skills [18–20]. There is a clear rationale for examining whether the impact of HL on mental health varies by HL trajectory type, characterised by age at onset and persistence of HL.

This study aimed to investigate the relationship between childhood HL, age at HL onset, HL trajectories, and risk of depressive symptoms and self-harm using information from a nationally representative prospective observational cohort of UK children followed from age 9 months to 14 years. Additionally, we examined the relationship of HL with peer victimisation, given that this presents a potential mediator of the mental health outcomes under examination. However, we did not undertake a mediation analysis since we could not assume that peer victimization preceded depressive symptoms in time because information on peer victimization and depressive symptoms were both collected at age 14 years only.

## Methods

### Participants

Participants came from the Millennium Cohort Study (MCS), a prospective, observational UK study of children from 19,244 families. Eligible children were those born in the UK between 2000 to 2002, who were alive and living in the UK at 9 months of age. The sample was selected from the Child Benefit register using a clustered, stratified design with oversampling to ensure representation of children living in areas of high poverty or, in England, with large ethnic-minority populations. The first study visit occurred when children were aged 9 months with follow-up at ages 3, 5, 7, 11, and 14 years. All visits were conducted in the home by trained interviewers using computer questionnaires [21].

**Fig. 1:**
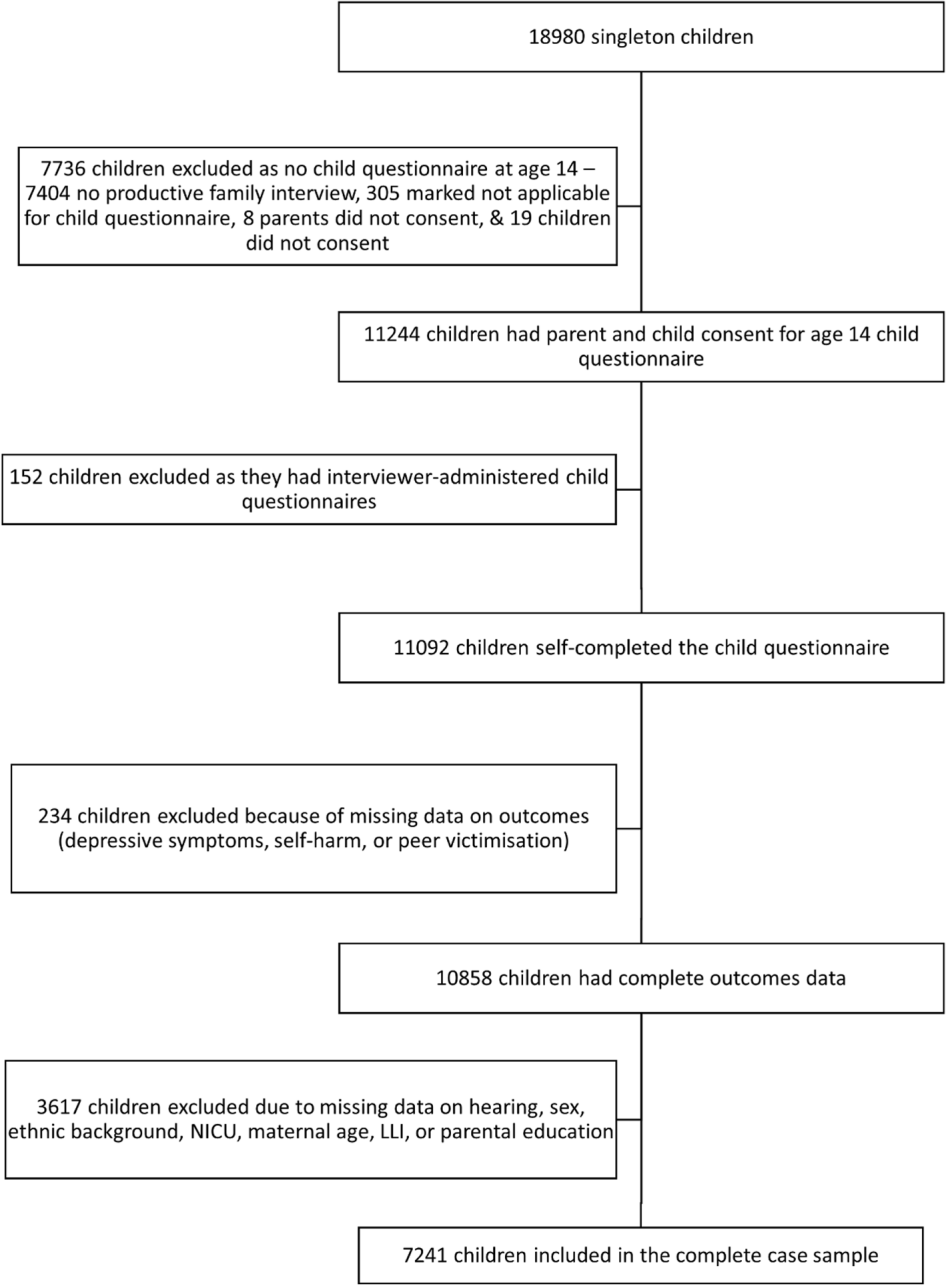
Participant flow diagram

Initial complete-case analyses involved 7241 singleton children with complete data on HL, peer victimisation, depressive symptoms, self-harm, and all sources of confounding (variable definitions given below). Multiple imputation addressed missing data in the exposure and sources of confounding, which increased the study population for analysis to 10,858 singleton children with available data on peer victimisation, depressive symptoms, and self-harm. Multiple births were excluded (*n =* 538 children). In the sample of singleton children, the most common reason for exclusion was attrition by or at age 14 years (*n* = 7404 children). In total, 3617 children had complete data on outcomes but were missing other data (Fig. 1).

### Variables

All outcomes were self-reported at age 14 years. Depressive symptoms were reported using the Short Mood and Feelings Questionnaire (SMFQ)*,* a clinically validated questionnaire for 6–17 year olds. The SMFQ consists of 13 statements about depressive symptoms over the preceding 2 weeks [22]. Answers to each statement were scored as: “not true” = 0, “sometimes true” = 1, “true” = 2, or missing = “don’t know” or “don’t want to answer”. Scores were summed (for a total ranging from 0 to 26) [23] and dichotomised into “low or no” levels of depressive symptoms (total score less than 8) versus “high” (total score 8 or more). This is a standard cut-off used for the SMFQ, although a sensitivity analysis was also carried out to examine results when using a cut-off of 12, another commonly cited cut-off in the literature [22, 24]. Cronbach’s alpha for the SMFQ questions in this study population was 0.93.

With respect to self-harm, children answered “yes” or “no” to the question, “In the past year have you hurt yourself on purpose in any way?”. Other responses (“don’t know”, “don’t want to answer”, or “not applicable”) were classed as missing.

A single binary variable indicating whether a child “never or rarely” experienced peer victimisation or was “regularly victimised by peers” was derived from two questions: “How often do other children hurt you or pick on you on purpose?” and “How often have other children sent you unwanted or nasty emails, texts or messages or posted something nasty about you on a website?”. The first question was deemed to reflect “in-person” victimisation, and the second “cyber-victimisation”. The “regularly victimised” category included children responding to either question with “most days” or “about once a week”. The “never or rarely” category included children reporting that both types of peer victimisation occurred “about once a month” or less frequently (i.e. every few months, less often, or never). This variable was classed as missing if children responded to either question with “don’t know” or “don’t want to answer”, or there was no response.

HL was reported by parents at all ages except age 14 years, when it was self-reported by children. At age 9 months, parents reported whether the child had any HL detected by a hearing test (this would usually refer to the Health Visitor Distraction Test, the standard screening test used at the time in this age group). At ages 3, 5, 7, and 11 years, parents were asked whether the child had “ever had” ear or hearing problems. If so, they reported the type of problem (coded using ICD-10 codes by trained coders) and any expected or received treatment. At age 14 years, children were asked whether they had hearing difficulties or used hearing aids. Children were then assigned to a HL presence category, defined as:

- Any HL: parent- or self-report of HL at any sweep. Between ages 3 and 11 years, HL was defined as having a HL-related ICD-10 code (e.g. H90: sensorineural or conductive hearing loss) or hearing-related treatment (hearing aids, cochlear implants, or grommets) (File S1)
- No HL: no HL reported at any sweep. This includes children with ear problems that were not reported to be hearing related between 3 and 11 years

Children were also allocated to one of six mutually exclusive HL trajectory groups as follows:

- No HL: no HL across all sweeps
- HL with onset and resolution in infancy: HL reported at age 9 months only with no ear problems or HL reported after this point
- Other early onset HL with resolution: HL reported with onset (first reported problem) by, or at, age 5 years. No HL reported at age 14 years and did not fit into the above “onset and resolution in infancy” group
- Early HL without resolution: HL reported with onset by, or at, age 5 years and HL reported at age 14 years
- Late onset HL with resolution: HL reported with onset after age 5 years and no HL at age 14 years
- Late HL without resolution: HL reported with onset after age 5 years and HL reported at age 14 years

Early onset HL was defined as onset of HL by, or at, age 5 years because this represents a sensitive period for communication development, thus HL onset at an earlier age may impact on communication more than HL onset at a later age [25]. Resolution by age 14 years was considered to understand whether the HL persisted into adolescence. HL definition was not restricted by aetiology, severity, mechanism, or expected natural history. Children with missing data on hearing at any sweep were classed as missing.

The following sources of confounding were selected based on expected associations between HL and one or more of the outcomes:

- Child’s sex: boys or girls, based on parent report at their first interview and questions about puberty at age 14 years
- Child’s ethnic background: white or non-white, based on self-report at age 14 years or last parent report where self-report was missing
- Child’s Neonatal Intensive Care Unit (NICU) admission: yes or no, based on parent report at first interview
- Parental education: equivalent national vocational qualification (NVQ) level 1–5 (coded by the Millennium Cohort Study team), or other, based on parent report at age 14 years, or latest data at other sweeps if missing (except ages 5 or 7 years as this was categorised differently). Marked as “other” if equivalent NVQ level unclear. NVQ levels 1 and 2 are equivalent to UK GCSE-level qualifications only (i.e. 15–16 years of age) and NVQ level 4 is equivalent to undergraduate degree level.
- Maternal age at birth of the child: completed years, as reported by parents at age 9 months
- Child’s limiting longstanding illness additional to HL (LLI): not present at any sweep or present at one or more sweeps after age 9 months, based on parent report across all sweeps. Examples of categories are: vision or mobility problems, as well as social and behavioural issues

### Statistical methods

Firstly, the distribution of variables in the study population was explored. Next, the relationship of HL presence with each source of confounding was examined using univariable logistic regression models. The associations of HL trajectory type with the sources of confounding were analysed using multinomial logistic regression models.

Relationships between HL and peer victimisation, depressive symptoms, and self-harm were examined by fitting univariable, then multivariable, logistic regression models with the latter adjusting for sources of confounding. The models included either HL presence or HL trajectory type as the exposure. Interaction terms between sex and HL presence were included where statistically significant, but not fully explored in the HL trajectory models due to the small numbers in some trajectory groups.

Sensitivity analyses using the complete-case dataset were carried out. These entailed fitting models excluding children with HL reported at age 14 years only; children with “other ear problems” only (i.e. ear problems not confirmed as hearing-related at any sweep); and children with any indication of glue ear based on ICD-10 codes or treatment with grommets. Additionally, the relationship between HL and SMFQ score was examined using a higher cut-off (12 instead of 8).

Multiple imputation with chained equations addressed missing data in the exposure and sources of confounding for children with completely observed outcomes (*n* = 10,858) [26]. HL presence and trajectory were imputed (3571 children had missing values) along with NICU (44 missing), LLI (2217 missing), parental education (2 missing), and maternal age (32 missing). Continuous variables were imputed using predictive mean matching (with 10 nearest neighbours), binary variables with logistic regression, nominal variables with multinomial logistic regression, and ordinal variables with ordinal logistic regression. Sex and ethnicity were not imputed as there were no missing values in children with observed outcomes, however, these variables were used as predictors in the imputation. We stratified the dataset by sex when imputing as interactions were explored in the analyses.

Other predictors used for imputation included survey design factors, the outcomes, autism, parental distress, young person’s independence, whether the child had any close friends at age 14 years, parent-reported Strengths and Difficulties peer problems and prosocial subscale scores at age 14 years, and language skills at age 14 years.

We created 35 imputed datasets given that approximately 33% of data were missing [26]. We confirmed that after 10 iteration cycles per imputed dataset there were no trends or instabilities in imputed values of maternal age. Additionally, imputed values were examined for plausibility and their distribution compared with observed values.

All results were adjusted for the two-stage stratified cluster sampling design and the probability of not having a productive interview at age 14 years. Pooled estimates across imputed datasets were calculated using Rubin’s rules [26]. Results reported below use imputed data unless indicated.

Analyses were carried out using StataSE 15 (StataCorp, College Station, TX) and R version 3.4.1 (R Foundation for Statistical Computing, Vienna, Austria) [27]. R packages used were tidyverse [28], haven [29], readxl [30], mice [26], miceadds [31], and VIM [32].

## Results

### Descriptive characteristics

**Fig. 2:**
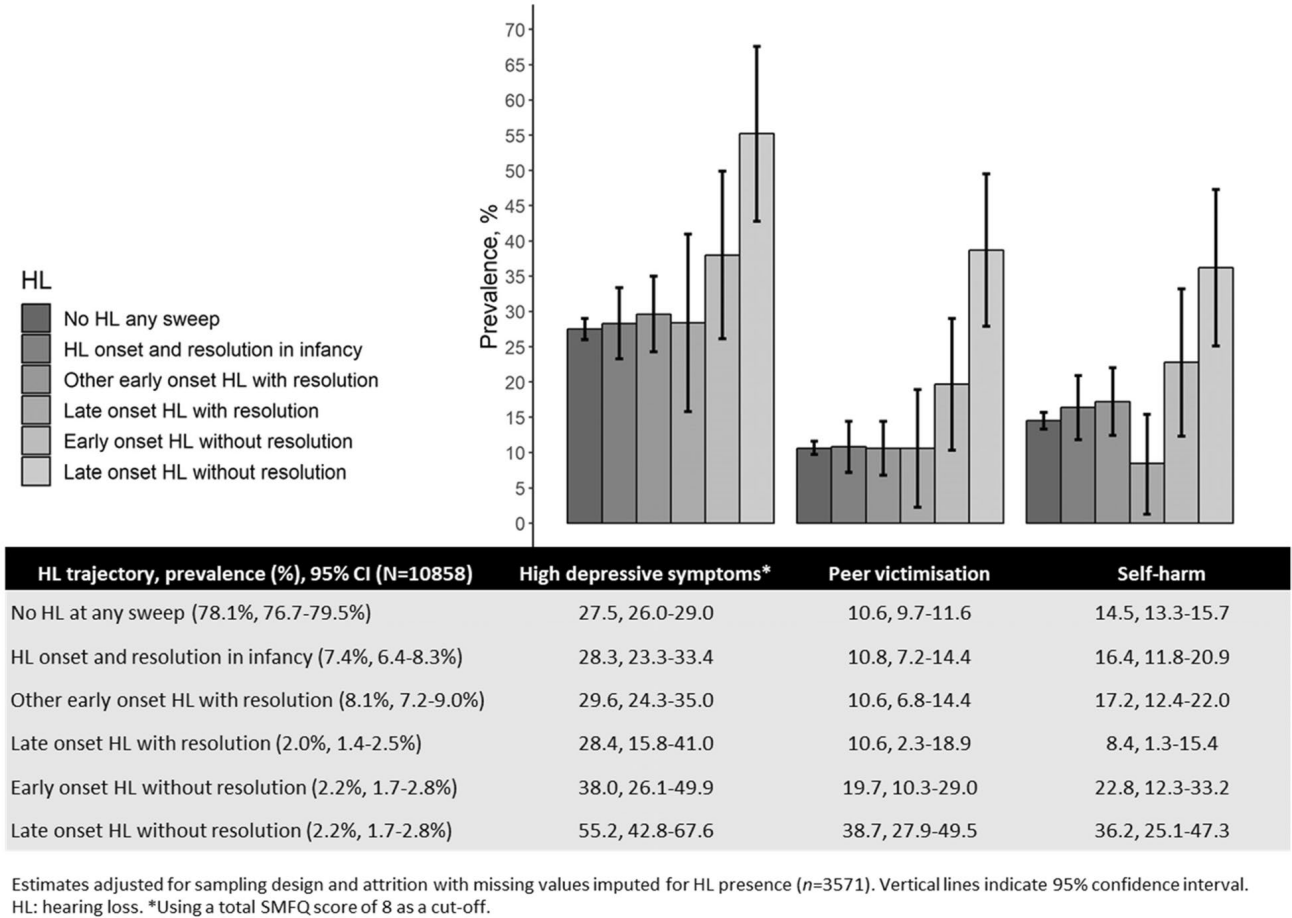
Prevalence of HL trajectories and outcomes

Approximately one fifth of children (21.9, 95% confidence interval [CI]: 20.5–23.3%) experienced HL at least once during the first 14 years of life, with the majority of HL occurring in early childhood and resolving by age 14 years (Fig. 2). Hearing aids and cochlear implants were reported for fewer than 1% of children at each sweep of data collection.

High levels of depressive symptoms (henceforth referred to only as “depressive symptoms”) were reported by 3003 children (28.6, 95% CI: 27.4–29.9%), peer victimisation by 1166 children (11.5, 95% CI: 10.7–12.3%) and self-harm in the last year by 1582 children (15.4, 95% CI: 14.4–16.4%). All outcomes were more frequent in children with HL and varied by HL trajectory (Fig. 2).

Over half of the study population were boys (51.6%; 95% CI: 50.5–52.7%) and the majority were from a white ethnic background (80.9%;95% CI: 78.0–83.8%). Almost 20% (19.2, 95% CI: 18.0–20.5%) had LLI at one or more sweeps and 8.5% (95% CI: 7.8–9.2%) had been admitted to NICU. Mean maternal age was 27.9 (95% CI: 27.6–28.2) years and the majority of parents had at least NVQ level 2 education. Univariable logistic regression models indicated that children with any HL were less likely to be girls (odds ratio [OR]: 0.74, 95% CI: 0.63–0.88) or of non-white ethnic background (OR: 0.75, 95% CI: 0.57–0.99), and more likely to have LLI (OR: 1.74, 95% CI: 1.41–2.14). Boys were over-represented in the group of children with HL in infancy only or HL with early onset and resolution. Children in all HL trajectory groups, except HL in infancy only, had higher odds of LLI than children without HL (Fig. S1 and Table S1).

### Depressive symptoms

There were increased odds of high depressive symptoms in children with any HL (adjusted OR: 1.32, 95% CI: 1.09–1.60; Table 1 and Table S2). Compared to children without HL, only children in the trajectory group with late onset HL that did not resolve had significantly higher odds of depressive symptoms (adjusted OR: 3.21: 1.91–5.40; Table 2 and Table S3). Similar findings were identified when using a higher cut-off for high depressive symptoms. Most sensitivity analyses confirmed these findings, with the exception of excluding children with HL at age 14 when the odds of higher depressive symptoms in children with HL decreased to 1.18 (95% CI: 0.99–1.40). There was no significant interaction by sex in the model.

**Table 1:** Association between HL presence and each outcome based on multivariable logistic regression models

*N* = 10,858 children	High depressive symptoms OR (95% CI)	Peer victimisation OR (95% CI)	Self-harm OR (95% CI)
HL presence and sex	n/a		n/a
Boys, HL absent		Reference	
Boys, HL present vs absent		1.02 (0.71–1.46)	
Girls vs boys, HL absent		0.89 (0.73–1.09)	
HL presence in girls vs HL presence in boys		1.81 (1.10–2.99)	
Any HL, present	1.32 (1.09–1.60)	n/a	1.41 (1.12–1.78)
Sex, girls	2.88 (2.56–3.24)	n/a	3.31 (2.85–3.85)
Ethnic background, non-white	0.76 (0.64–0.91)	0.65 (0.50–0.85)	0.65 (0.51–0.83)
NICU admission	1.29 (1.08–1.55)	1.01 (0.78–1.32)	0.99 (0.78–1.26)
Parental education	*p* = 0.33	*p* = 0.43	*p* = 0.24
NVQ1	Reference	Reference	Reference
NVQ2	1.26 (0.98–1.62)	1.26 (0.86–1.83)	1.04 (0.77–1.41)
NVQ3	1.18 (0.89–1.55)	1.24 (0.82–1.86)	0.85 (0.61–1.18)
NVQ4	1.08 (0.83–1.39)	1.37 (0.94–2.00)	0.87 (0.65–1.18)
NVQ5 (highest)	1.11 (0.84–1.45)	1.08 (0.73–1.60)	0.79 (0.57–1.09)
Other	1.17 (0.88–1.55)	1.15 (0.79–1.68)	0.89 (0.61–1.29)
LLI at one or more sweeps	1.54 (1.30–1.82)	1.50 (1.21–1.87)	1.57 (1.28–1.92)
Maternal age, per year increase	0.99 (0.98–1.00)	0.98 (0.97–1.00)	0.99 (0.98–1.00)
Baseline odds	0.23 (0.16–0.34)	0.15 (0.09–0.26)	0.11 (0.07–0.18)

Estimates are adjusted for sampling design and attrition, and include imputed data for missing values (0 for sex and ethnic background, 2 for parental education, 32 for maternal age, 44 for NICU, 2217 for LLI, and 3571 for HL). *CI* confidence interval, *HL* hearing loss, *LLI* limiting longstanding illness additional to HL, *n/a* not applicable as not included in the model, *NICU* neonatal intensive care unit, *NVQ* national vocational qualification (levels 1–2 equivalent to education to 15–16 years of age, level 3 to 18 years of age, level 4 to undergraduate degree and level 5 to postgraduate education), *OR* odds ratio

**Table 2:** Association between HL trajectory and each outcome based on multivariable logistic regression models

*N* = 10,858 children	High depressive symptoms OR (95% CI)	Peer victimisation OR (95% CI)	Self-harm OR (95% CI)
HL trajectory	*p* = 0.001	*p* < 0.001	*p* < 0.001
No HL at any sweep	Reference	Reference	Reference
HL onset and resolution in infancy	1.15 (0.87–1.52)	1.03 (0.69–1.52)	1.29 (0.90–1.84)
Other early onset HL with resolution	1.16 (0.87–1.54)	0.92 (0.60–1.40)	1.29 (0.90–1.87)
Late onset HL with resolution	1.07 (0.54–2.11)	0.87 (0.35–2.13)	0.52 (0.20–1.38)
Early onset HL without resolution	1.40 (0.81–2.44)	1.86 (1.01–3.40)	1.50 (0.80–2.81)
Late onset HL without resolution	3.21 (1.91–5.40)	4.88 (2.99–7.97)	3.22 (1.96–5.29)
Sex, girls	2.87 (2.55–3.22)	1.01 (0.86–1.19)	3.28 (2.82–3.82)
Ethnic background, non-white	0.77 (0.64–0.91)	0.66 (0.51–0.86)	0.66 (0.52–0.83)
NICU admission	1.30 (1.09–1.56)	1.05 (0.80–1.37)	0.99 (0.78–1.27)
Parental education	*p* = 0.33	*p* = 0.50	*p* = 0.24
NVQ1	Reference	Reference	Reference
NVQ2	1.25 (0.97–1.62)	1.25 (0.85–1.84)	1.03 (0.76–1.40)
NVQ3	1.17 (0.89–1.55)	1.23 (0.81–1.86)	0.84 (0.60–1.17)
NVQ4	1.07 (0.82–1.39)	1.36 (0.92–2.01)	0.86 (0.64–1.16)
NVQ5 (highest)	1.11 (0.84–1.47)	1.09 (0.73–1.63)	0.78 (0.56–1.09)
Other	1.16 (0.87–1.55)	1.13 (0.76–1.67)	0.88 (0.60–1.29)
LLI at one or more sweeps	1.53 (1.30–1.81)	1.49 (1.19–1.87)	1.57 (1.28–1.92)
Maternal age, per year increase	0.99 (0.98–1.00)	0.98 (0.97–1.00)	0.99 (0.98–1.00)
Baseline odds	0.23 (0.16–0.34)	0.15 (0.08–0.26)	0.11 (0.07–0.18)

Estimates are adjusted for sampling design and attrition, and include imputed data for missing values (0 for sex and ethnic background, 2 for parental education, 32 for maternal age, 44 for NICU, 2217 for LLI, and 3571 for HL). *CI* confidence interval, *HL* hearing loss, *LLI* limiting longstanding illness additional to HL, *NICU* neonatal intensive care unit, *NVQ* national vocational qualification, *OR* odds ratio

### Peer victimisation

A significant interaction between HL presence and sex was detected. The final model, including this interaction, indicated that boys and girls without HL experienced similar odds of peer victimisation. Boys with HL did not have higher odds of peer victimisation, however, the adjusted OR for the association between HL and peer victimisation in girls versus boys was 1.81 (95% CI: 1.10–2.99). This suggests that HL is more strongly associated with peer victimisation in girls (Table 1 and Table S4). Sensitivity analyses and stratified analyses confirmed these conclusions (in stratified analyses, the adjusted OR of peer victimisation was 1.81 [1.29–2.55] times higher in those with HL for girls, and in in boys: 1.05 [0.73–1.51]).

In terms of HL trajectory type, children with HL that did not resolve had higher adjusted odds of peer victimisation, regardless of age at onset (adjusted ORs were 1.86 [95% CI: 1.01–3.40] and 4.88 [2.99–7.97] for early and late onset HL without resolution, respectively; Table 2 and Table S5). Sensitivity analyses confirmed these conclusions.

### Self-harm

Adjusted odds of self-harm were higher in children with HL (1.41, 95% CI: 1.12–1.78; Table 1 and Table S6) with no evidence of an interaction by sex. Sensitivity analyses confirmed these conclusions. The adjusted odds of self-harm were higher in children with late onset HL that did not resolve (3.22, 95% CI: 1.96–5.29; Table 2 and Table S7), with no other differences in odds by HL trajectory when compared to children without HL. Sensitivity analyses confirmed these conclusions.

## Discussion

### Key results

Childhood HL is associated with higher odds of self-reported adolescent depressive symptoms, self-harm, and – in girls only - peer victimisation. Children with late onset HL without resolution were more likely to experience all outcomes, thus timing of onset and persistence appear to be important in the association between HL and mental health outcomes. These findings build upon previous studies by identifying specific mental health disorders associated with HL and demonstrating differences in the likelihood of poor mental health by HL trajectory. This is the first study to examine risk of self-harm and HL and the findings provide the foundations for further research to understand the causal pathways underlying these associations.

Our findings suggest that children with HL may be at higher risk of depressive symptoms, notably those with persistent HL of later childhood onset. Previous studies have reported conflicting findings on this topic. This may reflect the use of selective samples and restrictive HL definitions, meaning that previous results are only reflective of a small subgroup of children with HL [5–14, 16, 17]). Our analyses using robust statistical methods and a prospective study design confirm the association of childhood HL with self-reported depressive symptoms.

We also found that girls with HL may be at higher risk of peer victimisation than those without HL. This association was not found in boys. This might be explained by differences in the type of peer victimisation by sex, for instance physical versus social [33]. Few studies have examined the association of HL with subsequent peer victimisation and we did not identify any published studies assessing whether this varies by child sex [14, 34–36].

To our knowledge this study is the first to examine and confirm a higher risk of self-harm in children with HL. Furthermore, we found that children with late onset HL without resolution had the highest odds of self-harm. This research requires replication in other settings to confirm the validity of our findings. Our results also indicate a need to consider HL trajectory (in terms of timing of onset and resolution) in future studies of HL and mental health.

### Strengths and limitations

This study used data from a large population-based cohort study, which ensured generalisability of the results. Moreover, the data were collected prospectively across childhood, thus reducing the risk of recall bias and facilitating identification of HL trajectories. The use of self-reported mental health outcomes in this study represents a strength given that self-report is often more accurate than reports by parents or other informants for internalising symptoms such as depressive symptoms on [37–39]. Finally, during analyses, we adjusted for the non-random survey design and utilised multiple imputation to ensure that findings are generalisable to the UK population of young people.

Potential limitations are the lack of objective measures of HL in the MCS and self-reported HL should not be considered as a direct proxy for objective measures, however self-reported HL at age 14 should capture perceived HL while previous parent reports will not. We cannot exclude the possibility that the experience of depressive symptoms or peer victimisation might have influenced self-reporting of HL by young people. Approximately 75% of children in the “late onset HL without resolution” only self-reported HL at age 14 years and had no parental report of HL. This may represent progressive or later-onset HL, or young people may report mild or moderate HL which they perceive as a problem, while parents only report more severe HL. In future studies, it would be useful to capture both parental and self-report of HL, as well as objective measures, to understand how reporting of HL by parents and young people compares. Owing to the lack of objective measures, the type or severity of HL could not be examined. This is important to address in further research to understand whether the different trajectories of HL have different severities, which may impact on mental health. The identified prevalence of HL in this study (21.9% of young people experienced HL by age 14 years) is slightly higher than that estimated in a recent systematic review (17.9% young people had unilateral/bilateral HL > 15 dB HL) [40], but seems plausible given that the present study included all HL from age 9 months to 14 years and used a subjective measure of HL. Furthermore, future studies could also examine whether treatment for HL modifies the association between HL and peer victimisation or mental health. The self-harm measure was also limited in that there was only one question and this was not validated against other measures. However, the prevalence of self-harm was similar to that found in a systematic review of self-harm prevalence [41]. Additionally, whilst we adjusted for potential sources of confounding, it is possible that residual confounding remains, which could bias estimates. Finally, the data were collected prior to the introduction of newborn screening which may limit external validity to children born after 2002.

## Conclusion

Our findings imply a potential higher risk of depressive symptoms, self-harm, and peer victimisation in children with HL relative to those without HL. This association appears most marked for children with persistent HL with onset in later childhood. Our findings highlight the need for increased support for families and children to prevent these outcomes and enable children with HL to achieve good mental health. These findings merit replication in other settings and qualitative research to understand the impact of persistent HL occurring in later childhood. Further analyses should explore factors involved in the association between HL and the outcomes, including type of LLI, age at HL diagnosis, treatment type, school type, and communication method. Formal causal analyses are needed to characterise the pathways and mechanisms that may underlie the identified associations. This includes analysing whether peer victimisation may act as a mediator on a pathway between HL and depressive symptoms or self-harm.

## Supplementary Information

**Additional file 1: File S1.** Classification of hearing loss. **Figure S1.** Descriptive characteristics by hearing loss trajectory. **Table S1.** Descriptive characteristics by hearing loss presence. **Table S2.** Logistic regression results for depressive symptoms and hearing loss presence. **Table S3.** Logistic regression results for depressive symptoms and hearing loss trajectories. **Table S4.** Logistic regression results for peer victimisation and hearing loss presence. **Table S5.** Logistic regression results for peer victimisation by hearing loss trajectory. **Table S6.** Logistic regression results for self-harm and hearing loss presence. **Table S7.** Logistic regression results for self-harm and hearing loss trajectories.

## Data Availability

Data from the MCS are available from the UK Data Archive [42–47]. Millennium Cohort Study datasets used in this study are available on the UK Data Service website (https://beta.ukdataservice.ac.uk/datacatalogue/series/series?id=2000031). The data identifier code is SN 2000031.

## Abbreviations

CI: Confidence intervals
HL: Hearing loss
LLI: Limiting longstanding illness
MCS: Millennium Cohort Study
NICU: Neonatal intensive care unit
NVQ: National Vocational Qualification
OR: Odds ratio
SMFQ: Short Mood and Feelings Questionnaire
