# Supplementary material for: The association between childhood hearing loss and self-reported peer victimisation, depressive symptoms, and self-harm: longitudinal analyses of a prospective, nationally representative cohort study
Source: BMC Public Health. 2022 May 25;22:1045. doi: 10.1186/s12889-022-13457-6 (PMC9131522; doi:10.1186/s12889-022-13457-6)
Supplement: Supplementary file 1 — Additional file 1: File S1. Classification of hearing loss. Figure S1. Descriptive characteristics by hearing loss trajectory. Table S1. Descriptive characteristics by hearing loss presence. Table S2. Logistic regression results for depressive symptoms and hearing loss presence. Table S3. Logistic regression results for depressive symptoms and hearing loss trajectories. Table S4. Logistic regression results for peer victimisation and hearing loss presence. Table S5. Logistic regression results for peer victimisation by hearing loss trajectory. Table S6. Logistic regression results for self-harm and hearing loss presence. Table S7. Logistic regression results for self-harm and hearing loss trajectories. [file 12889_2022_13457_MOESM1_ESM.docx]

#### File S1. Classification of hearing loss

Between the ages of 3 and 11 years, data on each child’s hearing status were obtained by asking their parents a series of questions about whether their child had experienced any ear or hearing problems. If parents reported problems, they were probed for detail on the type of problem (coded to ICD-10 codes by trained coders) and whether the child had received, or was due to receive, any treatment.

A large number of different ICD-10 codes and treatments were reported and multiple problems could be reported per child at each sweep. We developed categorisation systems to assign each child with a reported ear or hearing problem to a single mutually exclusive ICD-10 code group and treatment group. In the case of multiple reported problems or treatment at a single sweep, children were assigned to a group based on their most significant problem (in order of the groups in the below tables).

ICD-10 code groups

| Group | Type | ICD-10 | Meanings |
| --- | --- | --- | --- |
| 1 | Hearing Loss (HL) | H90 | Conductive and Sensorineural HL |
|  |  | H91 | Other Deafness |
|  |  | Q16 | Congenital Malformation Of Ear Causing Impairment Of Hearing |
|  |  | Z45 | Adjustment And Management Of Implanted Hearing Device |
|  |  | Z46 | Fitting And Adjustment Of Other Devices (includes Hearing Aids) |
|  |  | Z96 | Presence Of Ontological And Audiological Implants |
|  |  | Z97 | Wearing Hearing Aid |
| 2 | Not HL | H60 | Otitis Externa |
|  |  | H61 | Other Disorders |
|  |  | H65 | Non-suppurative Otitis Media (including Glue Ear) |
|  |  | H66 | Suppurative/Unsp. Otitis Media |
|  |  | H67 | Otitis Media Classified Elsewhere |
|  |  | H68 | Obstructed/Stricture Of Eustachian Tube |
|  |  | H69 | Other Disorders Of Eustachian Tube |
|  |  | H70 | Mastoiditis & Related |
|  |  | H72 | Perforated Tympanic Membrane |
|  |  | H73 | Other Disorders Of Tympanic Membrane |
|  |  | H74 | Other Disorder Of Middle Ear And Mastoid |
|  |  | H81 | Disorders Of Vestibular Function |
|  |  | H83 | Other Disease Of Inner Ear |
|  |  | H92 | Otalgia And Effusion |
|  |  | H93 | Other Disorders Of Ear, Not Elsewhere Classified |
|  |  | H95 | Postprocedural Disorders Of Ear And Mastoid Process, Not Elsewhere Classified |
|  |  | Q17 | Other Congenital Malformations Of Ear |
|  |  | Q35 | Cleft Palate |
|  |  | T16 | Foreign Body In Ear |
|  |  | A87 | Viral Meningitis |
|  |  | B07 | Viral Warts |
|  |  | B34 | Viral Infection, Unsp. Site |
|  |  | B58 | Toxoplasmosis |
|  |  | B99 | Other And Unspecified Infectious Diseases |
|  |  | D84 | Other Immunodeficiencies |
|  |  | F60 | Specific Personality Disorders |
|  |  | F80 | Specific Speech Articulation Disorder |
|  |  | F92 | Mixed Disorders Of Refraction And Accommodation |
|  |  | F98 | Other Behavioural And Emotional Disorders With Onset Usually Occurring In Childhood And Adolescence |
|  |  | G03 | Meningitis |
|  |  | G40 | Epilepsy |
|  |  | G47 | Sleep Disorder |
|  |  | G58 | Other Mononeuropathies |
|  |  | H45 | Disorders Of Vitreous Body And Globe In Diseases Classified Elsewhere |
|  |  | H57 | Other Disorders Of Eye And Adnexa |
|  |  | I51 | Complications And Ill-Defined Descriptions Of Heart Disease |
|  |  | J00 | Cold |
|  |  | J02 | Acute Pharyngitis |
|  |  | J03 | Acute Tonsillitis |
|  |  | J21 | Acute Bronchiolitis |
|  |  | J22 | Other Acute Lower Respiratory Infections |
|  |  | J30 | Vasomotor And Allergic Rhinitis |
|  |  | J31 | Chronic Rhinitis, Nasopharyngitis And Pharyngitis |
|  |  | J32 | Chronic Sinusitis |
|  |  | J34 | Other Disorders Of Nose And Nasal Sinuses |
|  |  | J35 | Chronic Diseases Of Tonsils And Adenoids |
|  |  | J39 | Other Diseases Of Upper Respiratory Tract |
|  |  | J45 | Asthma |
|  |  | K52 | Other Noninfective Gastroenteritis And Colitis |
|  |  | L02 | Cutaneous Abscess, Furuncle And Carbuncle |
|  |  | L29 | Pruritus |
|  |  | L30 | Other Dermatitis |
|  |  | L72 | Follicular Cysts Of Skin And Subcutaneous Tissue |
|  |  | L91 | Hypertrophic Disorders Of Skin |
|  |  | Q02 | Microcephaly |
|  |  | Q18 | Other Congenital Malformations Of Face And Neck |
|  |  | Q82 | Other Congenital Malformations Of Skin |
|  |  | Q87 | Other Specified Congenital Malformation Syndromes Affecting Multiple Systems |
|  |  | Q89 | Other Congenital Malformations, Not Otherwise Classified |
|  |  | R04 | Haemorrhage From Respiratory Passages |
|  |  | R11 | Nausea And Vomiting |
|  |  | R21 | Rash And Non-Specific Skin Eruption |
|  |  | R22 | Localized Swelling, Mass And Lump Of Skin And Subcutaneous Tissue |
|  |  | R27 | Other Lack Of Coordination |
|  |  | R42 | Dizziness |
|  |  | R47 | Speech Disturbances, Not Elsewhere Classified |
|  |  | R56 | Convulsions, Not Elsewhere Classified |
|  |  | R59 | Enlarged Lymph Nodes |
|  |  | R62 | Lack Of Expected Normal Physiological Development |
|  |  | R63 | Symptoms And Signs Concerning Food And Fluid Intake |
|  |  | S04 | Injury Of Cranial Nerves |
|  |  | S05 | Injury Of Eye And Orbit |
|  |  | Y40 | Systemic Antibiotics |
|  |  | H52 | Disorders Of Refraction And Accommodation |
|  |  | P07 | Disorders Related To Short Gestation And Low Birth Weight, Not Elsewhere Classified |
|  |  | R17 | Unspecified Jaundice |
|  |  | R69 | Unknown And Unspecified Causes Of Morbidity |
|  |  | T78 | Adverse Effects, Not Elsewhere Classified |
|  |  | T88 | Other Complications Of Surgical And Medical Care, Not Elsewhere Classified |
|  |  | W44 | Foreign Body Entering Into Or Through Eye Or Natural Orifice |
|  |  | Z00 | General Examination And Investigation Of Persons Without Complaint And Reported Diagnosis |
|  |  | Z01 | Other Special Examinations And Investigations Of Persons Without Complaint And Reported Diagnosis |
|  |  | Z03 | Medical Observation And Evaluation For Suspected Diseases And Conditions |
|  |  | Z04 | Examination And Observation For Other Reasons |
|  |  | Z13 | Special Screening Examination For Other Diseases And Disorders |
|  |  | Z40 | Prophylactic Surgery |
|  |  | Z41 | Procedures For Purposes Other Than Remedying Health State |
|  |  | Z50 | Care Involving Use Of Rehabilitation Procedures |
|  |  | Z75 | Problems Related To Medical Facilities And Other Health Care |
|  |  | 899 |  |
|  |  | XXX |  |
|  |  |  | No Code Given But Child Has Problem Recorded |

Treatment code groups

| Group | Type | Includes |
| --- | --- | --- |
| 1 | HL Treatment | Hearing Aid or Cochlear Implants |
| 2 | Not HL treatment | Grommets, Antibiotics, Adenoidectomy, Tonsillectomy, Wax Removal Or “Cleaning Out Of The Ears”, Nasal Spray, Decongestant, Balloon Treatment, Antihistamines, or Ear Drops, Operation (Not Further Classified), Check-Ups/Tests, Pain-Killers, Surgery To Pin Ears Back, Homeopathic Medicines, ‘Drops Nsf’, ‘Spray Nsf’, Other Medicines, Physiotherapy, Vents, Stents, Any Vague Answer Or ‘Waiting For Appointment’, ‘Don’t Know’, or No Answer But Problem Reported, No Treatment |

Fig S1. Descriptive characteristics by hearing loss trajectory


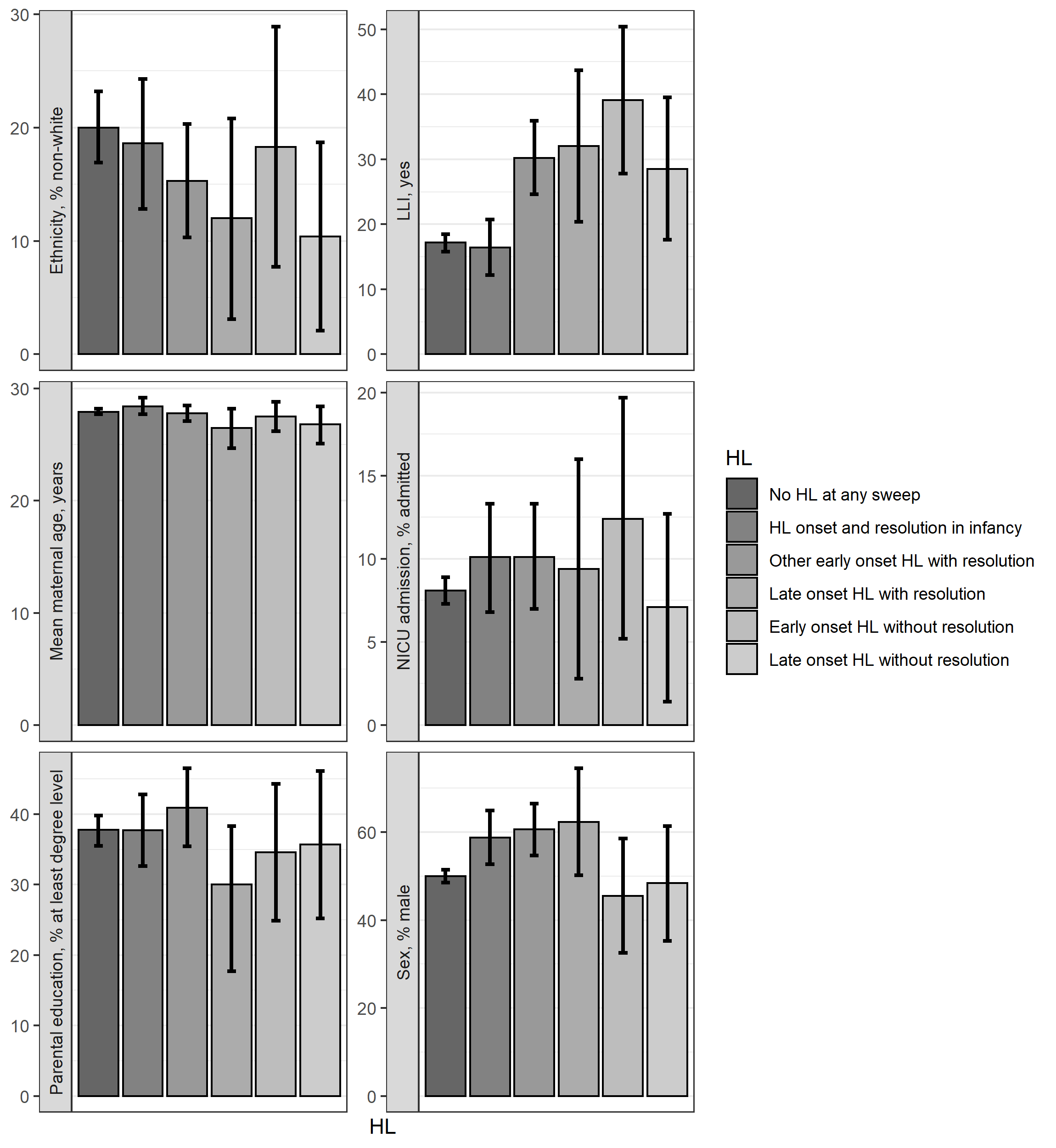


Estimates are adjusted for sampling design and attrition and include imputed data for children with missing values (0 for sex and ethnic background, 2 for parental education, 32 for maternal age, 44 for NICU, 2217 for LLI, and 3571 for HL). Vertical lines indicate 95% confidence intervals for each estimate. HL: hearing loss; LLI: limiting longstanding illness additional to HL; NICU: neonatal intensive care unit.

| ***N*=10858 children** | **Any HL** | **No HL** | **Total** | **Odds ratio for any HL vs. no HL, 95% CI** | ***p-*value** |
| --- | --- | --- | --- | --- | --- |
| **Child sex, %, 95% CI**  Boys  Girls | 57.4, 53.9-60.8  42.6, 39.2-46.1 | 50.0, 48.5-51.4  50.0, 48.6-51.5 | 51.6, 50.5-52.7  48.4, 47.3-49.5 | Reference  0.74, 0.63-0.88 | <0.01 |
| **Child ethnic background, %, 95% CI**  White  Non-white | 84.1, 80.3-87.9  15.9, 12.1-19.7 | 80.0, 76.8-83.1  20.0, 16.9-23.2 | 80.9, 78.0-83.8  19.1, 16.2-22.0 | Reference  0.75, 0.57-0.99 | 0.04 |
| **Child LLI, %, 95% CI**  No LLI  LLI at one or more sweeps | 73.5, 70.0-77.0  26.5, 23.0-30.0 | 82.8, 81.5-84.2  17.2, 15.8-18.5 | 80.8, 79.5-82.0  19.2, 18.0-20.5 | Reference  1.74, 1.41-2.14 | <0.001 |
| N**ICU admission, %, 95% CI**  No  Yes | 90.0, 88.0-92.0  10.0, 8.0-12.0 | 91.9, 91.1-92.7  8.1, 7.3-8.9 | 91.5, 90.8-92.2  8.5, 7.8-9.2 | Reference  1.26, 0.95-1.66 | 0.10 |
| **Highest parental education, %, 95% CI**  NVQ1  NVQ2  NVQ3  NVQ4  NVQ5 (highest)  Other | 6.6, 4.7-8.5  26.8, 23.3-30.3  13.7, 11.5-16.0  27.5, 24.8-30.3  10.0, 8.3-11.6  15.4, 12.0-18.8 | 7.0, 6.1-7.9  26.1, 24.4-27.8  14.1, 13.0-15.1  29.0, 27.4-30.7  8.6, 7.8-9.5  15.1, 13.3-16.9 | 6.9, 6.1-7.7  26.3, 24.9-27.6  14.0, 13.1-14.9  28.7, 27.2-30.2  8.9, 8.1-9.7  15.2, 13.6-16.7 | Reference  1.10, 0.73-1.66  1.04, 0.71-1.53  1.01, 0.72-1.42  1.23, 0.82-1.85  1.09, 0.69-1.71 | 0.79 |
| **Maternal age, mean, 95% CI** | 27.7, 27.3-28.2 | 27.9, 27.6-28.2 | 27.9, 27.6-28.2 | 0.99, 0.98-1.01, per year increase | 0.42 |

Table S1. Descriptive characteristics by hearing loss presence

Estimates are adjusted for sampling design and attrition and include imputed data for children with missing values (0 for sex and ethnic background, 2 for parental education, 32 for maternal age, 44 for NICU, 2217 for LLI, and 3571 for HL). CI: confidence interval; HL: hearing loss; LLI: limiting longstanding illness additional to HL; NICU: neonatal intensive care unit; NVQ: national vocational qualification. Odds ratios and *p*-values are from univariable logistic regression with any HL as the outcome.

**Table S2. Logistic regression results for depressive symptoms and hearing loss presence**

|  | High versus low depressive symptoms, OR (95% CI) | | | |
| --- | --- | --- | --- | --- |
|  | **Univariable analysis without imputation (*n*=7241)** | **Univariable analysis with imputation (*N*=10858)** | **Multivariable analysis without imputation (*n*=7241)** | **Multivariable analysis with imputation (*N*=10858)** |
| Any HL, present | 1.21 (1.03-1.42) | 1.27 (1.06-1.53) | 1.28 (1.09-1.50) | 1.32 (1.09-1.60) |
| Sex, girls | 2.77 (2.42-3.17) | 2.76 (2.46-3.09) | 2.87 (2.51-3.28) | 2.88 (2.56-3.24) |
| Ethnic background, non-white | 0.80 (0.67-0.96) | 0.73 (0.62-0.86) | 0.80 (0.66-0.98) | 0.76 (0.64-0.91) |
| NICU admission | 1.05 (0.83-1.33) | 1.25 (1.05-1.49) | 1.10 (0.88-1.38) | 1.29 (1.08-1.55) |
| Parental education  NVQ1  NVQ2  NVQ3  NVQ4  NVQ5 (highest)  Other | Wald test *p*=0.30  Reference  1.16 (0.82-1.63)  1.04 (0.74-1.47)  0.95 (0.69-1.32)  1.00 (0.71-1.42)  1.18 (0.79-1.77) | *p*=0.13  Reference  1.25 (0.97-1.61)  1.14 (0.87-1.50)  1.03 (0.80-1.33)  1.05 (0.80-1.38)  1.15 (0.86-1.54) | Wald test *p*=0.51  Reference  1.17 (0.83-1.64) 1.05 (0.73-1.47) 0.98 (0.71-1.36) 1.05 (0.74-1.49)  1.17 (0.78-1.76) | *p*=0.33  Reference  1.26 (0.98-1.62)  1.18 (0.89-1.55)  1.08 (0.83-1.39)  1.11 (0.84-1.45)  1.17 (0.88-1.55) |
| LLI at one or more sweeps | 1.49 (1.26-1.76) | 1.53 (1.30-1.80) | 1.53 (1.28-1.82) | 1.54 (1.30-1.82) |
| Maternal age, per year increase | 0.99 (0.97-1.00) | 0.99 (0.98-1.00) | 0.99 (0.98-1.00) | 0.99 (0.98-1.00) |
| Baseline odds | Variable | Variable | 0.26 (0.16-0.43) | 0.23 (0.16-0.34) |

Estimates are adjusted for sampling design and attrition and, where indicated, include imputed data for children with missing values (0 for sex and ethnic background, 2 for parental education, 32 for maternal age, 44 for NICU, 2217 for LLI, and 3571 for HL). Baseline odds are odds of high depressive symptoms if all variables are at baseline values. CI: confidence interval; HL: hearing loss; LLI: limiting longstanding illness additional to HL; NICU: neonatal intensive care unit; NVQ: national vocational qualification; OR: odds ratio.

**Table S3. Logistic regression results for depressive symptoms and** **hearing loss trajectories**

|  | High versus low depressive symptoms, OR (95% CI) | | | |
| --- | --- | --- | --- | --- |
|  | **Univariable without imputation (*n*=7241)** | **Univariable with imputation *(N*=10858)** | **Multivariable without imputation (*n*=7241)** | **Multivariable with imputation (*N*=10858)** |
| HL trajectory  No HL at any sweep HL onset & resolution in infancy Other early onset HL with resolution Late onset HL with resolution Early onset HL no resolution Late onset HL no resolution | Wald *p<*0.001  Reference 1.04 (0.81-1.33) 1.05 (0.83-1.33) 1.02 (0.62-1.67) 1.44 (0.84-2.45) 3.31 (2.18-5.04) | *p*<0.001  Reference  1.04 (0.80-1.36)  1.11 (0.84-1.46)  1.03 (0.55-1.95)  1.61 (0.95-2.72)  3.25 (1.94-5.45) | Wald *p*<0.001  Reference  1.14 (0.88-1.48)  1.13 (0.89-1.43)  1.10 (0.66-1.84)  1.26 (0.70-2.28)  3.36 (2.20-5.11) | *p*=0.001  Reference  1.15 (0.87-1.52)  1.16 (0.87-1.54)  1.07 (0.54-2.11)  1.40 (0.81-2.44)  3.21 (1.91-5.40) |
| Sex, girls | 2.77 (2.42-3.17) | 2.76 (2.46-3.09) | 2.86 (2.50-3.27) | 2.87 (2.55-3.22) |
| Ethnic background, non-white | 0.80 (0.67-0.96) | 0.73 (0.62-0.86) | 0.81 (0.66-0.99) | 0.77 (0.64-0.91) |
| NICU admission | 1.05 (0.83-1.33) | 1.25 (1.05-1.49) | 1.12 (0.89-1.40) | 1.30 (1.09-1.56) |
| Parental education  NVQ1  NVQ2  NVQ3  NVQ4  NVQ5 (highest)  Other | Wald *p*=0.30  Reference  1.16 (0.82-1.63)  1.04 (0.74-1.47)  0.95 (0.69-1.32)  1.00 (0.71-1.42)  1.18 (0.79-1.77) | *p*=0.13  Reference  1.25 (0.97-1.61)  1.14 (0.87-1.50)  1.03 (0.80-1.33)  1.05 (0.80-1.38)  1.15 (0.86-1.54) | Wald *p*=0.50  Reference  1.17 (0.83-1.65) 1.05 (0.74-1.49) 0.98 (0.70-1.37) 1.06 (0.74-1.51)  1.18 (0.78-1.78) | *p*=0.33  Reference  1.25 (0.97-1.62)  1.17 (0.89-1.55)  1.07 (0.82-1.39)  1.11 (0.84-1.47)  1.16 (0.87-1.55) |
| LLI at one or more sweeps | 1.49 (1.26-1.76) | 1.53 (1.30-1.80) | 1.52 (1.28-1.82) | 1.53 (1.30-1.81) |
| Maternal age, per year increase | 0.99 (0.97-1.00) | 0.99 (0.98-1.00) | 0.99 (0.98-1.00) | 0.99 (0.98-1.00) |
| Baseline odds | Variable | Variable | 0.26 (0.16-0.43) | 0.23 (0.16-0.34) |

Estimates are adjusted for sampling design and attrition and, where indicated, include imputed data for children with missing values (0 for sex and ethnic background, 2 for parental education, 32 for maternal age, 44 for NICU, 2217 for LLI, and 3571 for HL). Baseline odds are odds of high depressive symptoms if all variables are at baseline values. CI: confidence interval; HL: hearing loss; LLI: limiting longstanding illness additional to HL; NICU: neonatal intensive care unit; NVQ: national vocational qualification; OR: odds ratio.

**Table S4. Logistic regression results for peer victimisation and hearing loss presence**

|  | Peer victimisation, OR (95% CI) | | | |
| --- | --- | --- | --- | --- |
|  | **Univariable without imputation (*n*=7241)** | **Univariable with imputation (*N*=10858)** | **Multivariable without imputation (*n*=7241)** | **Multivariable with imputation (*N*=10858)** |
| Boys, HL absent  Boys, HL present vs absent  Girls vs boys, HL absent  HL presence in girls vs HL presence in boys^a^ | Reference  1.03 (0.76-1.39)  0.74 (0.60-0.92)  1.73 (1.05-2.83) | Reference  1.08 (0.76-1.54)  0.89 (0.73-1.09)  1.80 (1.09-3.00) | Reference  0.97 (0.72-1.32)  0.74 (0.60-0.91)  1.75 (1.06-2.90) | Reference  1.02 (0.71-1.46)  0.89 (0.73-1.09)  1.81 (1.10-2.99) |
| Ethnic background, non-white | 0.63 (0.48-0.82) | 0.63 (0.49-0.80) | 0.64 (0.48-0.84) | 0.65 (0.50-0.85) |
| NICU admission | 1.02 (0.72-1.43) | 1.10 (0.84-1.44) | 0.93 (0.65-1.32) | 1.01 (0.78-1.32) |
| Parental education  NVQ1  NVQ2  NVQ3  NVQ4  NVQ5 (highest)  Other | Wald test *p*=0.64  Reference  1.27 (0.77-2.10)  1.29 (0.76-2.18)  1.25 (0.75-2.11)  1.14 (0.68-1.92)  0.94 (0.53-1.67) | *p*=0.26  Reference  1.23 (0.85-1.78)  1.18 (0.79-1.76)  1.25 (0.87-1.81)  0.96 (0.65-1.42)  1.02 (0.72-1.46) | Wald test *p*=0.82  Reference  1.31 (0.78-2.19) 1.34 (0.79-2.29) 1.34 (0.79-2.28) 1.25 (0.74-2.13)  1.09 (0.61-1.96) | *p*=0.43  Reference  1.26 (0.86-1.83)  1.24 (0.82-1.86)  1.37 (0.94-2.00)  1.08 (0.73-1.60)  1.15 (0.79-1.68) |
| LLI at one or more sweeps | 1.51 (1.22-1.87) | 1.57 (1.27-1.95) | 1.48 (1.19-1.84) | 1.50 (1.21-1.87) |
| Maternal age, per year increase | 0.99 (0.98-1.01) | 0.98 (0.97-1.00) | 0.99 (0.97-1.01) | 0.98 (0.97-1.00) |
| Baseline odds | Varies by model | Varied by model | 0.14 (0.07-0.27) | 0.15 (0.09-0.26) |

Estimates adjusted for sampling design and attrition and, where indicated, include imputed data for children with missing values (0 for sex and ethnic background, 2 for parental education, 32 for maternal age, 44 for NICU, 2217 for LLI, and 3571 for HL). Baseline odds are odds of peer victimisation if all variables are at baseline values. CI: confidence interval; HL: hearing loss; LLI: limiting longstanding illness additional to HL; NICU: neonatal intensive care unit; NVQ: national vocational qualification; OR: odds ratio. ^a^Interaction term: ratio of OR for peer victimisation in girls with versus without HL, versus OR for peer victimisation in boys with versus without HL.

**Table S5. Logistic regression results for peer victimisation by hearing loss trajectory**

|  | Peer victimisation, OR (95% CI) | | | |
| --- | --- | --- | --- | --- |
|  | **Univariable without imputation (*n*=7241)** | **Univariable with imputations (*N*=10858)** | **Multivariable without imputation (*n*=7241)** | **Multivariable with imputations (*N*=10858)** |
| HL trajectory No HL at any sweep HL onset and resolution in infancy Other early onset HL with resolution Late onset HL with resolution Early onset HL without resolution Late onset HL without resolution | Wald *p*<0.001  Reference  1.04 (0.73-1.49)  0.94 (0.67-1.33)  0.88 (0.45-1.70)  1.88 (1.08-3.27)  4.95 (3.03-8.08) | *p*<0.001  Reference  1.02 (0.68-1.52)  0.99 (0.65-1.51)  0.96 (0.39-2.32)  2.04 (1.11-3.74)  5.31 (3.28-8.59) | Wald *p*<0.001 Reference 1.02 (0.72-1.46) 0.86 (0.61-1.21) 0.79 (0.40-1.56) 1.74 (0.99-3.04) 4.62 (2.79-7.67) | *p*<0.001  Reference  1.03 (0.69-1.52)  0.92 (0.60-1.40)  0.87 (0.35-2.13)  1.86 (1.01-3.40)  4.88 (2.99-7.97) |
| Sex, girls | 0.83 (0.69-1.01) | 1.02 (0.87-1.20) | 0.82 (0.68-1.00) | 1.01 (0.86-1.19) |
| Ethnic background, non-white | 0.63 (0.48-0.82) | 0.63 (0.49-0.80) | 0.65 (0.49-0.86) | 0.66 (0.51-0.86) |
| NICU admission | 1.02 (0.72-1.43) | 1.10 (0.84-1.44) | 0.96 (0.68-1.36) | 1.05 (0.80-1.37) |
| Parental education  NVQ1  NVQ2  NVQ3  NVQ4  NVQ5 (highest)  Other | Wald *p*=0.64  Reference  1.27 (0.77-2.10)  1.29 (0.76-2.18)  1.25 (0.75-2.11)  1.14 (0.68-1.92)  0.94 (0.53-1.67) | *p*=0.26  Reference  1.23 (0.85-1.78)  1.18 (0.79-1.76)  1.25 (0.87-1.81)  0.96 (0.65-1.42)  1.02 (0.72-1.46) | Wald p=0.80 Reference 1.31 (0.78-2.22) 1.35 (0.78-2.32) 1.34 (0.77-2.34) 1.27 (0.74-2.21) 1.08 (0.59-1.96) | *p*=0.50  Reference  1.25 (0.85-1.84)  1.23 (0.81-1.86)  1.36 (0.92-2.01)  1.09 (0.73-1.63)  1.13 (0.76-1.67) |
| LLI at one or more sweeps | 1.51 (1.22-1.87) | 1.57 (1.27-1.95) | 1.47 (1.17-1.84) | 1.49 (1.19-1.87) |
| Maternal age, per year increase | 0.99 (0.98-1.01) | 0.98 (0.97-1.00) | 0.99 (0.97-1.01) | 0.98 (0.97-1.00) |
| Baseline odds | Varies by model | Varied by model | 0.13 (0.06-0.25) | 0.15 (0.08-0.26) |

Estimates adjusted for sampling design and attrition and, where indicated, include imputed data for children with missing values (0 for sex and ethnic background, 2 for parental education, 32 for maternal age, 44 for NICU, 2217 for LLI, and 3571 for HL). Baseline odds are odds of peer victimisation if all variables are at baseline values. CI: confidence interval; HL: hearing loss; LLI: limiting longstanding illness additional to HL; NICU: neonatal intensive care unit; NVQ: national vocational qualification; OR: odds ratio.

**Table S6. Logistic regression results for self-harm and hearing loss presence**

|  | Self-harm, OR (95% CI) | | | |  |
| --- | --- | --- | --- | --- | --- |
|  | **Univariable without imputation (*n*=7241)** | **Univariable with imputations (*N*=10858)** | **Multivariable without imputation (n=7241)** | **Multivariable with imputations (*N*=10858)** | |
| Any HL, present | 1.27 (1.03-1.58) | 1.35 (1.08-1.69) | 1.35 (1.10-1.67) | 1.41 (1.12-1.78) | |
| Sex, girls | 3.13 (2.62-3.75) | 3.18 (2.74-3.70) | 3.26 (2.73-3.90) | 3.31 (2.85-3.85) | |
| Ethnic background, non-white | 0.66 (0.52-0.85) | 0.61 (0.49-0.77) | 0.69 (0.53-0.90) | 0.65 (0.51-0.83) | |
| NICU admission | 0.95 (0.71-1.26) | 0.99 (0.78-1.25) | 0.98 (0.74-1.29) | 0.99 (0.78-1.26) | |
| Parental education  NVQ1  NVQ2  NVQ3  NVQ4  NVQ5 (highest)  Other | Wald *p*=0.05  Reference  1.13 (0.79-1.62)  0.80 (0.52-1.23)  0.81 (0.56-1.17)  0.88 (0.60-1.30)  0.87 (0.54-1.40) | *p*=0.08  Reference  1.05 (0.78-1.42)  0.84 (0.61-1.17)  0.85 (0.64-1.14)  0.77 (0.56-1.05)  0.88 (0.61-1.25) | Wald *p*=0.07  Reference  1.13 (0.78-1.64)  0.79 (0.52-1.22)  0.83 (0.57-1.20)  0.91 (0.62-1.36)  0.87 (0.53-1.42) | *p*=0.24  Reference  1.04 (0.77-1.41)  0.85 (0.61-1.18)  0.87 (0.65-1.18)  0.79 (0.57-1.09)  0.89 (0.61-1.29) | |
| LLI at one or more sweeps | 1.51 (1.23-1.86) | 1.55 (1.28-1.88) | 1.56 (1.27-1.92) | 1.57 (1.28-1.92) | |
| Maternal age, per year increase | 0.99 (0.97-1.00) | 0.99 (0.98-1.00) | 0.99 (0.97-1.00) | 0.99 (0.98-1.00) | |
| Baseline odds | Varied by model | Varied by model | 0.12 (0.07-0.22) | 0.11 (0.07-0.18) | |

Estimates adjusted for sampling design and attrition and, where indicated, include imputed data for children with missing values (0 for sex and ethnic background, 2 for parental education, 32 for maternal age, 44 for NICU, 2217 for LLI, and 3571 for HL). Baseline odds are odds of self-harm if all variables are at baseline values. CI: confidence interval; HL: hearing loss; LLI: limiting longstanding illness additional to HL; NICU: neonatal intensive care unit; NVQ: national vocational qualification; OR: odds ratio.

**Table S7. Logistic regression results for self-harm and hearing loss trajectories**

|  | Self-harm, OR (95% CI) | | | |
| --- | --- | --- | --- | --- |
|  | **Univariable without imputation (*n*=7241)** | **Univariable with imputations (*N*=10858)** | **Multivariable without imputation (*n*=7241)** | **Multivariable with imputations (*N*=10858)** |
| HL trajectory No HL at any sweep HL onset and resolution in infancy Other early onset HL with resolution Late onset HL with resolution Early onset HL without resolution Late onset HL without resolution | Wald test *p*<0.001  Reference  1.12 (0.83-1.51)  1.19 (0.86-1.64)  0.44 (0.20-0.99)  1.59 (0.89-2.84)  3.13 (1.84-5.30) | *p*<0.001  Reference  1.15 (0.81-1.63)  1.22 (0.84-1.76)  0.52 (0.20-1.33)  1.72 (0.93-3.17)  3.34 (2.03-5.50) | Wald test *p*<0.001  Reference  1.24 (0.92-1.67)  1.30 (0.95-1.77)  0.47 (0.21-1.06)  1.40 (0.79-2.46)  3.06 (1.86-5.05) | *p*<0.001  Reference  1.29 (0.90-1.84)  1.29 (0.90-1.87)  0.52 (0.20-1.38)  1.50 (0.80-2.81)  3.22 (1.96-5.29) |
| Sex, girls | 3.13 (2.62-3.75) | 3.18 (2.74-3.70) | 3.24 (2.71-3.87) | 3.28 (2.82-3.82) |
| Ethnic background, non-white | 0.66 (0.52-0.85) | 0.61 (0.49-0.77) | 0.69 (0.53-0.90) | 0.66 (0.52-0.83) |
| NICU admission | 0.95 (0.71-1.26) | 0.99 (0.78-1.25) | 0.99 (0.75-1.30) | 0.99 (0.78-1.27) |
| Parental education NVQ1 NVQ2 NVQ3 NVQ4 NVQ5 (highest) Other | Wald test *p*=0.05  Reference  1.13 (0.79-1.62)  0.80 (0.52-1.23)  0.81 (0.56-1.17)  0.88 (0.60-1.30)  0.87 (0.54-1.40) | *p*=0.08  Reference  1.05 (0.78-1.42)  0.84 (0.61-1.17)  0.85 (0.64-1.14)  0.77 (0.56-1.05)  0.88 (0.61-1.25) | Wald test *p*=0.07  Reference  1.13 (0.78-1.64)  0.79 (0.51-1.21)  0.83 (0.57-1.20)  0.91 (0.61-1.36)  0.87 (0.53-1.43) | *p*=0.24  Reference  1.03 (0.76-1.40)  0.84 (0.60-1.17)  0.86 (0.64-1.16)  0.78 (0.56-1.09)  0.88 (0.60-1.29) |
| LLI at one or more sweeps | 1.51 (1.23-1.86) | 1.55 (1.28-1.88) | 1.55 (1.27-1.91) | 1.57 (1.28-1.92) |
| Maternal age, per year increase | 0.99 (0.97-1.00) | 0.99 (0.98-1.00) | 0.99 (0.97-1.00) | 0.99 (0.98-1.00) |
| Baseline odds | Variable | Variable | 0.12 (0.07-0.22) | 0.11 (0.07-0.18) |

Estimates adjusted for sampling design and attrition, where indicated, and include imputed data for children with missing values (0 for sex and ethnic background, 2 for parental education, 32 for maternal age, 44 for NICU, 2217 for LLI, and 3571 for HL). Baseline odds are odds of self-harm if all variables are at baseline values. CI: confidence interval; HL: hearing loss; LLI: limiting longstanding illness additional to HL; NICU: neonatal intensive care unit; NVQ: national vocational qualification; OR: odds ratio.
